# Devastating floods in South Asia: The inequitable repercussions of climate change and an urgent appeal for action

**DOI:** 10.1016/j.puhip.2023.100365

**Published:** 2023-02-09

**Authors:** Saleha Azeem, Huzaifa Ahmad Cheema, Abia Shahid, Firoj Al-Mamun, Sudhan Rackimuthu, Mohammad Ebad Ur Rehman, Mohammad Yasir Essar, Ka Yiu Lee

**Affiliations:** Department of Community Medicine and Public Health, King Edward Medical University, Lahore, Pakistan; Department of Community Medicine and Public Health, King Edward Medical University, Lahore, Pakistan; Department of Medicine, King Edward Medical University, Lahore, Pakistan; CHINTA Research Bangladesh, Savar, Dhaka, Bangladesh; Department of Public Health & Informatics, Jahangirnagar University, Savar, Dhaka, Bangladesh; Department of Public Health, University of South Asia, Dhaka, Bangladesh; Father Muller Medical College, Kankanady, Mangalore, Karnataka, India; Department of Medicine, Rawalpindi Medical University, Rawalpindi, Pakistan; Kabul University of Medical Sciences, Kabul, Afghanistan; Swedish Winter Sports Research Centre, Department of Health Sciences, Mid Sweden University, Östersund, Sweden

Owing to various meteorological disasters such as heavy monsoon rainfall and glacial melt through the summer season of 2022, large parts of several countries in South Asia have been underwater. Pakistan and Sri Lanka are among the worst-hit countries with regions of Afghanistan, Bangladesh, and India also suffering [1]. Floods in Pakistan have caused nearly 1700 casualties, with over 12,900 being injured, and have damaged more than 1.3 million houses displacing about 7.9 million people [1]. Similarly in Sri Lanka, floods have affected over 15,000 people across ten districts either in the form of displacement, casualties, or injuries [1]. In Bangladesh, multitudes of people have been left unsheltered with no basic amenities of life. This has exposed all those who are affected by the floods to life-threatening water-borne diseases [1]. Furthermore, countries like Pakistan and Sri Lanka have been severely affected due to sky-high inflation rates, lack of employment opportunities, and insufficient provision of basic facilities to all areas [2]. Pakistan's economy relies on heavily on agriculture, and the loss of agricultural land along with other essential raw materials is extremely dangerous, especially for rural communities. Crops that are critical to supplying food to Pakistan such as rice and wheat have been wiped out by floods. Since these crops are usually cultivated by rural communities, it has also cost them their livelihoods. The recent domestic political instability has further exacerbated the situation, adversely affecting the already ailing economy of Pakistan [2]. Consequently, worsening health service, loss of shelter, and a dearth of food and water supplies have been observed.

In 2022, Pakistan experienced three times higher precipitation than normal while some of its states had as much as 7–8 times higher which is an indicator of the drastic alteration in the global climate [3]. According to the Indian Meteorological Department, heavy rainfall has been 85% more frequent in the country since 2012 [4]. Several factors such as increased industrialization, deforestation, burning of fossil fuels, and pollution caused by vehicles are considered to be responsible for climate change [3].

Countries in the global north are responsible for most of the carbon dioxide emissions contributing to global warming. The USA, Europe, Russia, Japan, and Canada are collectively responsible for approximately 85% of excess global carbon dioxide emissions [5]. In addition, the targets of the 2021 UN Climate Change Conference (COP26) have not been met. Only 23 out of 200 countries that signed the Glasgow agreement at COP26 submitted a UN climate change progress report by the mentioned deadline ^6^. Although countries like Egypt, Indonesia, and Brazil improved their targets, albeit still inadequate, responses from countries that are considered prime culprits remain unseen. Countries in the global south such as India and Pakistan, however, are within their boundary fair shares [5]. Unfortunately, these countries have to face the maximum brunt of climate change such as that in the form of natural disasters.

Thus, countries in the global north must take proactive steps to reduce their emissions to an acceptable level as soon as possible. Due to a disproportionately large contribution to climate change, a climate debt is owed by developed countries to developing ones to make up for the damage they might have caused. It is entirely reasonable to ask countries with excess emissions to pay or provide support; such help is not charity or external aid but is merely climate justice for the disproportionate damage that they have caused. This help should not only extend to deliver immediate humanitarian relief and monetary aid but also make up for losses involving consumables, infrastructure, and livelihoods. This is essential to ensure that countries like Pakistan, Bangladesh, and Sri Lanka that have minimally contributed to climate change do not suffer inequitably.

The 2022 UN Climate Change Conference (COP27) that was held recently reached a historic agreement to establish a fund that finances the loss and damage faced by vulnerable countries. While this is a huge step towards dealing with the inequitable repercussions of climate change, it remains to be seen whether this agreement will actually be put into practice. The previous targets set by COP26 have also been not met which include but are not limited to achieving carbon neutrality, protecting communities, and mobilizing climate finances. Therefore, it is imperative to put into place a mechanism of action where all of these goals are brought to completion. Future conferences must also place more emphasis on long-term climate change policies that reduce emissions by advocating the widespread transition to renewable energy resources.

If not properly taken care of through urgent political action and financial commitments, climate change could lead to irreversible loss of human life. However, most governments are currently least interested in taking action and enforcing necessary parameters to combat climate change. Colonial structures that undervalue less wealthy nations must not be reinforced and a shift in global governance must be ushered in by holding respective world leaders and nations accountable for their actions. Education and public awareness on a mass level also need to be carried out to increase the knowledge of the harms associated with climate change, encouraging the use of more environment-friendly appliances. Large companies and businesses should also be provided with incentives to take on a more environment-friendly approach to making products.

## Competing interests

The authors declare to have no conflicts of interest and no financial interests related to the material of this manuscript.

## Funding

No financial support was received for this study.

## Ethics approval

No ethical approval was required for this study as it is a letter to the editor and not based on any human data.

## Consent

No consent was required for this study.

## Authors’ contributions

All authors contributed equally to the conception and design of this study, drafting and critical revision of the article, and gave final approval for it.

## References

[1] 2022 South asian floods [internet]. Wikipedia. Available from: https://en.wikipedia.org/wiki/2022_South_Asian_floods.

[2] Pande A. Economic crisis threatens Pakistan again [Internet]. https://www.gisreportsonline.com/r/pakistan-crisis-economy/.

[3] World Weather Attribution Climate change likely increased extreme monsoon rainfall, flooding highly vulnerable communities in Pakistan [Internet]. https://www.worldweatherattribution.org/climate-change-likely-increased-extreme-monsoon-rainfall-flooding-highly-vulnerable-communities-in-pakistan/.

[4] Climate Change Causing Excessive Rain, Floods in India: Experts | Nagpur News - Times of India.

[5] Hickel J. (2020 Sep). Quantifying national responsibility for climate breakdown: an equality-based attribution approach for carbon dioxide emissions in excess of the planetary boundary. Lancet Planet. Health.

